# Blood-brain barrier permeability imaging using perfusion computed tomography

**DOI:** 10.2478/raon-2014-0029

**Published:** 2015-03-25

**Authors:** Jernej Avsenik, Sotirios Bisdas, Katarina Surlan Popovic

**Affiliations:** 1 Institute of Radiology, University Medical Centre Ljubljana, Slovenia; 2 Department of Neuroradiology, Eberhard Karls University, Tubingen, Germany

**Keywords:** blood-brain barrier, permeability imaging, computed tomography, perfusion CT

## Abstract

**Background.:**

The blood-brain barrier represents the selective diffusion barrier at the level of the cerebral microvascular endothelium. Other functions of blood-brain barrier include transport, signaling and osmoregulation. Endothelial cells interact with surrounding astrocytes, pericytes and neurons. These interactions are crucial to the development, structural integrity and function of the cerebral microvascular endothelium. Dysfunctional blood-brain barrier has been associated with pathologies such as acute stroke, tumors, inflammatory and neurodegenerative diseases.

**Conclusions.:**

Blood-brain barrier permeability can be evaluated *in vivo* by perfusion computed tomography - an efficient diagnostic method that involves the sequential acquisition of tomographic images during the intravenous administration of iodinated contrast material. The major clinical applications of perfusion computed tomography are in acute stroke and in brain tumor imaging.

## Introduction

The blood-brain barrier (BBB) is the system of tightly regulated anatomical and biochemical mechanisms that protects the brain from harmful compounds in the peripheral circulation, supplies brain cells with nutrients, functions as a dynamic regulator of ion balance and filters harmful substances from the brain to the bloodstream.^1^,^2^ It also restricts the entering of T-lymphocytes, maintaining the immune-privileged status of the brain.^3^ The BBB primarily represents the selective diffusion barrier at the level of the cerebral microvascular endothelium. Capillary lumen is enclosed by a single endothelial cell, characterized by the presence of tight junctions (TJ), the absence of fenestrations, increased number of mitochondria and minimal pinocytic activity in comparison to peripheral endothelium. Pericytes are attached to the abluminal membrane of the endothelium and together they are enclosed by the basal lamina, which is contiguous with the plasma membrane of astrocyte end-feet.^2^ Under physiologic conditions, the BBB is relatively impermeable. However, in pathologic conditions such as neoplasm, inflammatory/infectious disease and ischemia, the BBB permeability (BBBP) is increased^4^ and the diffusion of molecules into the extravascular space is enhanced.^5^,^6^ The increased BBBP can be evaluated *in vivo* by means of perfusion computed tomography (PCT) imaging.^7^,^8^

## Blood-brain barrier cellular structures

### Brain microvasculature endothelial cells

Brain endothelial cells represent the essential component of the BBB, performing functions such as diffusion barrier, transport, signaling, leukocyte transport and osmoregulation.^1^ Functional polarity exists between the apical and basolateral surface of the endothelial cell, which is evident by asymmetrical distribution of various transport-related carriers and enzymes present in the luminal and abluminal membranes.^9^,^10^ Endothelial cells are connected at the point of junctional complex, comprised predominantly of TJs and adherent junctions.

TJs, the critical component of BBB, are complex structures of intracellular and trans-membrane proteins, bound to an active cytoskeleton. This structure enables the tightness, as well as preserves the capacity for rapid regulation and functional modulation.^9^ Three major trans-membrane protein components of TJs are occludins^11^, claudins^12^, and the group of immunoglobulin gene superfamily proteins, namely junctional adhesion molecules (JAMs)^13^ and the endothelial selective adhesion molecules (ESAMs).^14^ These molecules are connected to a group of intracellular proteins called membrane-associated guanylate kinases (MAGUK) which function as a cytoplasmic adaptor proteins.^1^,^9^ First order adaptor proteins are zonula occludens (ZO-1, ZO-2 and ZO-3) and Ca^2+^-dependent protein serine kinase^15^–^17^, while the second order adapter proteins include cingulin, afadin and function-associated coiled-coil protein (JACOP). Besides providing the structural support, these proteins also interact with a large number of signaling and regulatory molecules, enabling the regulation of BBB permeability through local chemical signals. In addition to tight junctions, endothelial cells are also joined by adherent junctions, composed of transmembrane protein VE-cadherin, connected to cytoskeleton via catenins.^9^

Tightness of the BBB is also provided on the enzymatic level. Numerous enzymes were found to be present in BBB elements in significantly higher concentrations than in peripheral vessels. These enzymes metabolize neuroactive blood-borne products and include γ-glutamyl transpeptidase, alkaline phosphatase, aromatic acid decarboxylase and cytochrome 450 enzymes.^9^,^18^

Various transport systems are also crucial for the proper functioning of BBB. For instance, carrier-mediated transport represents highly specific system that allows the selective transport of small molecules, such as amino-acids, hexoses, nucleosides, amines and vitamins.^9^,^18^ Intracellular pH of endothelial cells as well as the optimal ion gradient across the membrane are provided by ion transporters, namely the sodium pump, sodium-potassium-two chloride co-transporter, chloride-bicarbonate exchanger and the sodium-hydrogen exchanger.^18^ Active efflux systems such as ATP-binding cassette (ABC) transporters, the multidrug resistance transporter P-glycoprotein (P-gp) and the group of multidrug resistance-associated proteins (MDRs) prevent the passage of drugs and toxins across the BBB and facilitate the efflux of neuroactive solutes from brain to blood. The transport across BBB for larger molecules like transferrin, low density lipoprotein, IgG, insulin and insulin like growth factor is provided by receptor mediated transport called transcytosis. Finally, absorptive mediated endocytosis represents less selective form of transport, initiated by polycathionic molecules binding to negatively charged plasma membrane.^9^

### Astrocytes, pericytes and neurons

Interactions of endothelial cells with surrounding cells as well as the extracellular matrix are crucial to their development, structural integrity^1^ and function.^19^,^20^ Astrocytes are glial cells whose end feet cover over 99% of the outer surface of the BBB endothelium.^1^,^20^ Soluble factors released by astrocytes play important role in enhancing TJs, reducing gap junctional area^21^ and also regulating water and electrolyte metabolism in the brain.^22^

Pericytes contribute to the low paracellular permeability of the BBB, perform a regulatory role in brain homeostasis, participate in vascular development and maintenance and also represent the source of adult pluripotent stem cells. Moreover, contractile, immune, phagocytic and migratory functions of pericytes have been described.^20^

Temporally and spatially adjusted blood supply in accordance to metabolic requirements of neurons is provided by intense communication between neurons, astrocytes and BBB. In addition to direct innervation of endothelial cells, neurons can regulate the BBB function through induction of specific enzymes in response to metabolic needs.^20^

## Blood-brain barrier in pathology

BBB dysfunction can range from mild and transient TJ opening to chronic barrier breakdown and has been associated with pathologies such as ischemia, tumors, multiple sclerosis, Parkinson’s disease, Alzheimer’s disease, epilepsy, glaucoma and lysosomal storage diseases.^23^

Hypoxia is the end point in many disorders such as acute stroke, cardiac arrest, carbon monoxide poisoning, respiratory distress and rapid ascent to high altitude and leads to increased BBB permeability, edema and tissue damage.^3^ Early interventions to reduce long term disease progression and disability rely on efficient diagnostic methods to identify the site and extent of BBB disturbance.^23^,^24^

## Perfusion computed tomography for the evaluation of blood-brain barrier permeability

The advent of fast computed tomography (CT) scanners in the 1990’s, together with the development of sophisticated post-processing software, has made PCT a powerful tool for investigating pathophysiological processes in the human body.^25^ *In vivo* evaluation and quantitative analysis of brain perfusion by means of PCT has had considerable impact on patient care in the settings of severe head trauma, acute stroke, and cerebral tumors.^26^–^30^ The determination of tissue perfusion by PCT involves the intravenous injection of tracer and subsequential imaging to monitor the concentration of tracer in the tissue and a feeding artery as functions of time.^27^ One important advantage of CT is that the enhancement is linearly proportional to the concentration of tracer in the tissue.^25^ Serial CT scans start before the contrast agent arrives to determine the baseline and repeated scans are acquired until the tracer leaves the tissues. Subtraction of the baseline from each of the serial CT scans after the arrival of the contrast agent at the tissue gives the time-density curve (TDC) of the tissue.^25^,^31^ All the physiological information is obtained by mathematical analysis of the tissue TDC. These analyses are based on proposed ‘tracer kinetics’ models that describe the distribution of contrast in blood vessels and extravascular space of the tissue.^25^,^31^

### Permeability imaging: basic concepts

BBBP describes how easy it is for a tracer molecule to move between the intravascular and extravascular space across the BBB. It is defined as the bulk flow of a tracer normalized for surface area, concentration gradient, and time:

[Equation 1] $$\frac{dC_{\mathrm{tissue}}}{dt}=P\cdot S\cdot M\cdot (C_{\mathrm{plasma}}-C_{EES})$$

where *P* is the permeability (cm/s); *S,* the surface area per unit mass (cm^2^/g); *M,* the tissue mass (g); *C*_plasma_ - *C*_EES_, the concentration difference between plasma and extravascular extracellular compartment (mmol/cm^3^).^6^ Blood-brain barrier permeability (BBBP) can be expressed as the permeability surface area product (PS) or as transendothelial transfer constant (K).

PS represents the total diffusional flux across all capillaries and is measured in ml/min/100g of tissue. It can be interpreted as following: the unidirectional flux of solutes from blood plasma to interstitial space is equivalent to the complete transfer of all the solutes in PS ml of blood per minute to interstitial space.

Another parameter, frequently used in the setting of permeability imaging is called extraction fraction (E). E represents the fraction of solutes in arterial blood, with the potential to diffuse into extravascular space that actually becomes transferred from blood to interstitial space during a single passage of blood from the arterial end to the venous end of the capillaries of a tissue.^32^

Different permeability parameters can be calculated by measuring the leakage of an intravascular tracer into the extravascular space.^5^,^32^,^33^ In the normal brain parenchyma, BBB is intact and tightly regulated. PS is normally 0 for large hydrophilic molecules such as a peripherally injected iodinated contrast agent.^5^ As mentioned, many pathologic situations such as tumor, inflammatory/infectious disease, and ischemia can alter BBB integrity and allow the diffusion of fluid, blood or contrast molecules into the extravascular space.^5^,^6^

### Tracer kinetic analysis

The analysis of PCT data for the evaluation of BBB can be done by parametric fitting using tracer kinetic models.^27^ Compartmental modelling as exemplified by the Patlak model^33^ assumes instantaneous mixing within the compartments^27^ and has been used by many authors.^5^,^8^,^34^–^36^ Alternatively, distributed parameter model (DPM) as first proposed by Johnson and Wilson^37^ describes tracer concentration in the vascular compartment as a function of both time and position along the capillary^27^ and is generally considered more accurate for the assessment of BBBP.^7^,^27^,^38^

### Compartmental modelling: Patlak model

A compartment is defined as a well-mixed space where the concentration is spatially uniform within the volume of distribution. In addition, the out flux at any outlet must be directly proportional to the concentration of tracer.^39^

The Patlak model is a unidirectional 2-compartment model that calculates BBBP via linear regression.^5^ Following injection into the blood stream, contrast agent will pass into the extravascular space at a rate that is characterized by transendothelial transfer constant K.^25^ The theoretical basis of imaging K is the Patlak graphical analysis^33^, which assumes that the injected contrast agent is distributed in two well-mixed compartments: the intravascular (blood) and the extravascular compartment. At any given time, a voxel of tissue will contain both intravascular and extravascular contrast agent. Assuming that during the time interval 0–t there is virtually no return of contrast agent from the extravascular to blood space, the total concentration of contrast agent in the tissue at time t, can be expressed as:

[Equation 2] $$Q(t)=V_{b}C_{a}(t)+K\int_{0}^{t}C_{a}(u)du$$

where Q(t) is the tissue enhancement at time t; C_a_(t) is the arterial enhancement at time t; and V_b_ is the distribution volume, which is typically considered to be equal to the cerebral blood volume (CBV) in the considered region of interest. In Equation 2, the first term on the right side describes the intravascular component of enhancement and the second term describes the extravascular component. The graphical analysis of the Patlak model divides both sides of Equation 2 by C_a_(t) to give the following equation, describing the Patlak plot:^25^,^34^

[Equation 3] $$\frac{Q(t)}{C_{a}(t)}=V_{b}+K\frac{\int_{0}^{t}C_{a}(u)du}{C_{a}(t)}$$

In this equation, the ratio of Q(t) to C_a_(t) is plotted on the y-axis and is called “apparent distribution volume”. The ratio of the integral of C_a_ to C_a_(t), which is plotted on the x-axis, is called “normalized plasma integral”. The slope of a regression line fit to the linear part of the Patlak plot is an approximation of K at that time. This value represents the amount of accumulated tracer in relation to the amount of tracer that has been available in plasma and is a measurement of BBBP expressed in mL /100 g/min. The y-axis intercept is equal to the V_b_ or CBV.^34^

Theoretic model of blood-brain exchange, described by Patlak *et al*.^33^,^34^, is relatively simple and frequently applied model to quantify BBBP from PCT data.^5^,^34^ It assumes the unidirectional transfer of a tracer from a reversible (arterial) compartment to an irreversible extravascular space (brain parenchyma) for a certain period of time.^34^,^40^ Transfer of tracer is assumed to be unidirectional when a steady-state phase is reached between reversible compartments (intravascular space and the blood-brain barrier complex). However, such a steady-state phase can only occur after the initial rapid changes in tracer concentration have subsided, so the arterial concentration decreases slowly enough for the tissue compartment to follow. Recent data suggest that only the delayed phase of the PCT acquisition (and not the first-pass) respects the assumptions of the Patlak model and that BBBP measurements extracted from first-pass PCT data overestimate BBBP values obtained from the delayed phase.^34^

The assumption that back-flux from extravascular into intravascular can be neglected during early times depends on the relative magnitude of blood flow (F) and the capillary permeability surface area product (PS).^25^ Permeability (P) is related to the diffusion coefficient of contrast agent in the assumed water-filled pores of the capillary endothelium. The diffusion flux of contrast agent across the capillary endothelium is dependent on both the diffusion coefficient and the total surface area of the pores or the PS product.^36^

The PS product has the same dimensions as F, and thus the ratio PS/F is dimensionless. PS is related to K by the following:

[Equation 4] K=EF

If PS/F < 1, then K ∼ PS. In normal cerebral vasculature, PS is negligible for all contrast agents presently in use.^36^ The relative magnitude of PS and F also determines E, according to the classic Renkin-Crone equation:^25^,^41^

[Equation 5] $$E=1-e^{\frac{-PS}{F}}$$

However, in the setting of various pathologic processes, it is doubtful whether the no back flux assumption will be valid in general. Another major drawback of compartmental models is a fact that F and E (PS) cannot be measured separately because they are determined together as K (EF). All information about the convective transport of solute along the capillaries is lost due to the assumption that intravascular space is a well-mixed compartment.^32^

### Distributed parameter model

**FIGURE 1.**
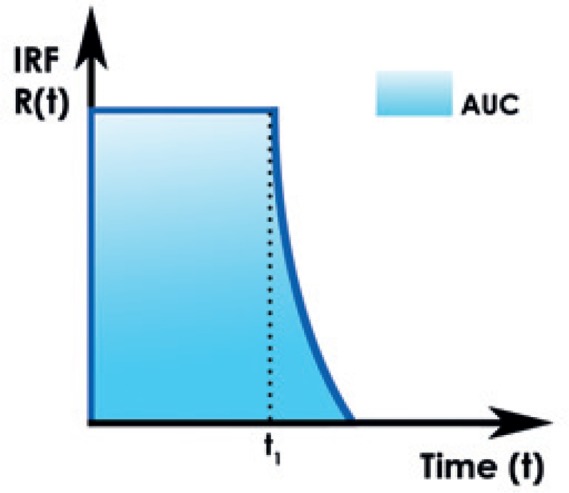
Impulse residue function (IRF). The IRF can be interpreted as the fraction of contrast medium that remains in the tissue as time evolves, following a bolus injection into arterial input. The duration of the plateau is the vascular transit time (t_1_). The area under the curve (AUC) is the mean transit time (MTT). As the Central Volume Principle states that the product of flow (F) and MTT is blood volume (CBV), the AUC of the flow corrected IRF (FR(t)) is the CBV. R(t) - the IRF at time t.^43^

Perfusion parameters can be derived from the impulse residue function (IRF). The IRF is a theoretical concept, *i.e*. a tissue TDC due to an idealized bolus injection of one unit of tracer into the arterial input.^31^,^42^ It describes the fraction of tracer that remains in the tissue as time evolves.^25^ Alternatively, it can be seen as the distribution of transit times in the tissue.^31^ For ease of calculation, the IRF is usually constrained in its shape to comprise a plateau followed by a single exponential decay (Figure 1).^42^ The duration of the plateau corresponds to the time interval during which all the injected contrast material remains in the capillary network.^28^ Contrast agent diffusion appears in the IRF as a residual enhancement that occurs after the initial impulse response and that decreases exponentially with time. The IRF is used to estimate the fraction of the mass of contrast agent arriving at the tissue that leaks into the extravascular space in a single passage through the vasculature, the extraction fraction (E).^25^,^36^

A mathematical process that uses arterial and tissue TDC to calculate IRF for the considered region of interest is called deconvolution. The height of the flow corrected IRF will give the tissue perfusion and the area under the curve (AUC) will determine the relative blood volume. This approach can be extended to include a measurement of capillary permeability by use of a distributed parameter model.^42^

In the Patlak model, the tracer concentration gradients within the vasculature are assumed to be zero.^32^ DPM on the other hand, takes the tracer concentration gradients within the vascular compartment into account, and may therefore allow more complete analysis of the perfusion parameters from a single PCT study.^25^,^44^ In contrast with compartmental models, it enables the separation of F and E (PS).^32^ Moreover, with the adiabatic approximation in time domain^32^,^45^, the model solution can be computed efficiently to generate functional maps of perfusion parameters.^25^

For the evaluation of BBBP, two compartment version of DPM has been used^7^ as it can be mathematically expressed in a separable form in time domain, each component describing a physiological process:

[Equation 6] $$\text{R}(\text{t})={\text{R}}_{\text{v}}(\text{t})+{\text{R}}_{\text{p}}(\text{t})$$

where R is IRF for vascular (v) and parenchymal (p) phase.^7^,^27^ For times, shorter than vascular transit time (duration of the plateau; t < t_1_), the vascular phase of the equation remains constant and is proportional to the total amount of tracer in the injected bolus.^7^,^44^ At t_1_, the unextracted tracer exits via outflowing blood, and the detector response registers the fraction of extracted tracer, given by E. Beyond the t_1_, the extracted tracer diffuses back into the blood and is cleared by outflowing blood, giving rise to a gradually decreasing parenchymal phase. The parameters that can be directly obtained from fitting experimental curves are F, t_1_, rate of transfer from intravascular to extravascular compartment (k_21_) and rate of transfer from extravascular to intravascular compartment (k_12_). With the DPM, E can be formally given by:^7^,^27^,^44^

[Equation 7] $$E(t_{1})=1-e^{-k_{21}t_{1}}$$

which is a function of the vascular transit time t_1_. This expression for E implies that, for two capillaries with the same outflow (extravasation) rate k21, the fraction of extracted tracer in the first-pass would be larger for the capillary with the longer transit time. The rate constant k_21_ can then be expressed as the ratio of the PS and fractional vascular volume (v_1_): k_21_ = ϱPS/v_1_. Since v_1_ can be estimated by v_1_ = ϱFt_1_, the PS could then be estimated as PS = k_21_v_1_/ϱ, and the latter equation reverts to the classic Renkin-Crone equation (Equation 5).^7^,^27^

## Clinical applications of BBBP imaging

The major clinical applications of PCT are in acute stroke and in brain tumor imaging.^31^

### Acute stroke

**FIGURE 2.**
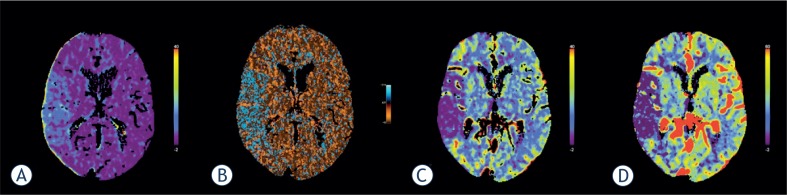
Perfusion computed tomography in acute stroke. Parametric maps show increased blood-brain barrier permeability values **(A, B)** in the right middle cerebral artery territory. The main advantage of Patlak’s analysis is its conceptual simplicity **(A)**. On the other hand, distributed parameter model takes the tracer concentration gradients within vasculature into account and may allow more complete analysis of kinetic parameters **(B)**. The delineation of ischaemic area is clearly recognized on blood flow **(C)** and blood volume **(D)** parametric maps.

PCT can be used to demonstrate elevated BBBP as an indicator of ischemia-induced vascular damage.^35^ Severe ischemia can alter BBB integrity and allow the diffusion of fluid, blood, or contrast molecules into the interstitium. A nonzero PS represents this diffusion quantitatively, and its functional color map can be generated by dedicated software (Figure 2).^5^ Ischemia or reperfusion induced damage to the BBB may lead to hemorrhagic transformation (HT) and poor clinical outcome independent of thrombolytic therapy.^7^ Symptomatic HT and malignant edema are feared complications in patients with acute ischemic stroke and occur 10 times more frequently in tPA-treated versus placebo-treated patients.^35^ Permeability analysis by means of PCT with DPM, proved to be an efficient tool for predicting HT in acute ischemic stroke.^7^ Another study has shown 100% sensitivity and 79 % specificity of admission BBBP imaging (using delayed acquisition PCT and Patlak model) in predicting symptomatic HT and malignant edema in acute ischemic stroke.^35^

### Brain tumor imaging

The development of a tumor blood supply through the process of angiogenesis is essential for the growth of tumors and also determines the ability of tumors to metastasize.^31^ Hypoxia or hypoglycemia that occurs in rapidly growing tumors increases the expression of vascular endothelial growth factor (VEGF), which is a potent permeability factor.^36^ Newly formed vessels are immature and have increased permeability to macromolecules due to large endothelial cell gaps, incomplete basement membrane, and absent smooth muscle.^36^,^46^

**FIGURE 3.**
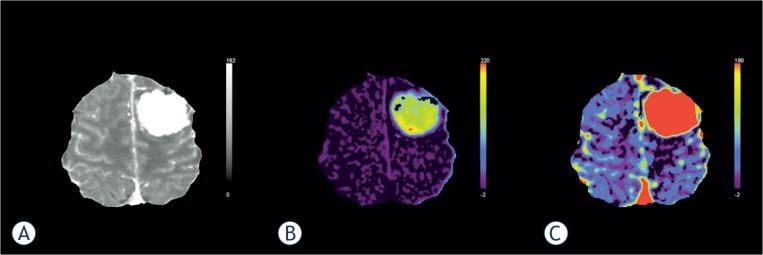
Perfusion computed tomography in brain tumours. Tracer kinetic analysis was performed in a patient with a large tumour in left cerebral hemisphere **(A)**, using Patlak model. The tumour tissue presents with significantly higher permeability values, indicating the immature leaky tumour vessels **(B)**. Unlike the blood volume parametric map **(C)**, permeability imaging also shows some local heterogeneity of tumour tissue.

Altered permeability of the newly formed tumor vessels can be effectively assessed by the PS and E parametric maps, which offer the additional advantage of tumor segmentation and delineation from surrounding healthy tissue (Figure 3).^47^ Both compartmental and distributed parameter modelling for contrast transport and exchange have been developed to quantify tissue F, CBV, MTT and permeability parameters.^32^ Significant difference in PS was found between low grade (WHO grade II) and high grade (WHO III or IV) glioma.^48^ Recent data even suggest that perfusion parameters, especially PS, can be used to differentiate grade III from grade IV glioma.^36^ PCT therefore provides useful information for glioma grading and has the potential to significantly impact clinical management of cerebral gliomas.^48^

## Conclusions

The BBB is tightly regulated system, performing functions such as diffusion barrier, transport, signaling and osmoregulation. In the normal brain parenchyma, BBB is intact and impermeable for large molecules such as iodinated contrast agent. In pathologic situations such as neoplasm, inflammatory/infectious disease, ischemia and some neurodegenerative disorders, the BBBP is altered and the diffusion of fluid, blood or contrast molecules into the extravascular space is enhanced. BBBP can be *in vivo* evaluated by PCT, which uses different mathematical models to calculate physiological information from raw data. An efficient method to identify and quantify the extent of BBB disturbance allows early intervention to reduce the long term disability in some patients. To date, the major clinical applications of PCT have been in acute stroke and in brain tumor imaging.
